# Accuracy evaluation of hand-eye calibration techniques for vision-guided robots

**DOI:** 10.1371/journal.pone.0273261

**Published:** 2022-10-19

**Authors:** Ikenna Enebuse, Babul K. S. M. Kader Ibrahim, Mathias Foo, Ranveer S. Matharu, Hafiz Ahmed

**Affiliations:** 1 Centre for Future Transport and Cities, Coventry University, Coventry, United Kingdom; 2 School of Mechanical, Aerospace and Automotive Engineering, Coventry University, Coventry, United Kingdom; 3 School of Engineering, University of Warwick, Coventry, United Kingdom; 4 Nuclear Futures Institute, Bangor University, Bangor, United Kingdom; University of Hull, UNITED KINGDOM

## Abstract

Hand-eye calibration is an important step in controlling a vision-guided robot in applications like part assembly, bin picking and inspection operations etc. Many methods for estimating hand-eye transformations have been proposed in literature with varying degrees of complexity and accuracy. However, the success of a vision-guided application is highly impacted by the accuracy the hand-eye calibration of the vision system with the robot. The level of this accuracy depends on several factors such as rotation and translation noise, rotation and translation motion range that must be considered during calibration. Previous studies and benchmarking of the proposed algorithms have largely been focused on the combined effect of rotation and translation noise. This study provides insight on the impact of rotation and translation noise acting in isolation on the hand-eye calibration accuracy. This deviates from the most common method of assessing hand-eye calibration accuracy based on pose noise (combined rotation and translation noise). We also evaluated the impact of the robot motion range used during the hand-eye calibration operation which is rarely considered. We provide quantitative evaluation of our study using six commonly used algorithms from an implementation perspective. We comparatively analyse the performance of these algorithms through simulation case studies and experimental validation using the Universal Robot’s UR5e physical robots. Our results show that these different algorithms perform differently when the noise conditions vary rather than following a general trend. For example, the simultaneous methods are more resistant to rotation noise, whereas the separate methods are better at dealing with translation noise. Additionally, while increasing the robot rotation motion span during calibration enhances the accuracy of the separate methods, it has a negative effect on the simultaneous methods. Conversely, increasing the translation motion range improves the accuracy of simultaneous methods but degrades the accuracy of the separate methods. These findings suggest that those conditions should be considered when benchmarking algorithms or performing a calibration process for enhanced accuracy.

## Introduction

Robots are leading the way in today’s growing need for automation and improved efficiency in the industry. This stems from their high level of repeatability, large payload capability and speed of operation. The International Federation of Robotics (IFR) predicted over 1.7 million new industrial robots’ deployments in 2021 [1], and many of these successes can be found in applications such as chicken deboning in the food industry [2–4], drug manufacturing in the pharmaceutical industry [5,6], and aircraft engine construction in the aerospace industry [7–9]. During the deployment of robots for automation, the level of autonomy given to a robot may vary depending on application and required flexibility. For some applications, like in the production of some specialised parts, robots are usually programmed to carry out a specific sequence of tasks repetitively with little or no variation. In these applications the velocity, acceleration, and direction of motions are predetermined and fed to the robot. In other applications, the robot is given more autonomy during its operation. This flexibility enables the robot to sense and react to its environment dynamically based on feedback from its sensors. For example, for more precise guidance, a vision sensor or camera can be attached to the robot to provide information for obstacle detection and avoidance, dynamic position acquisition of target for accurate tracking etc. The feedback from the vision sensor is used to improve the control implementation of the robot. These are called vision-guided-robots (VGR).

Generally, the configuration of the robot and camera can be one of two forms, namely, eye-to-hand configuration, and eye-in-hand configuration. In the eye-to-hand configuration, the camera is mounted in a fixed position, providing a fixed field of view throughout the entire robot operation. On the other hand, in the eye-in-hand configuration, the camera is attached directly on the robot such that new images can be acquired by changing the field of view of the camera through the robot motion. However, the robot can only perceive the 3D world based on its own base frame. For a robot to obtain an accurate estimate of the 3D position and orientation of a part relative to its own base within the work volume, it is necessary to know the relative position and orientation between the hand and the robot base, between the camera and the hand, and between the object and the camera. These three tasks require the calibration of robot [10,11], camera [12,13], and robot hand-to-camera (hand-eye) [14,15] to obtain the necessary accuracy. Robot calibration is needed because, even though robots have very good repeatability, they are poor when it comes to absolute accuracy, due to inherent differences between the ideal and actual kinematic parameters. This can be as a result of manufacturing and assembly tolerances, geometry of the robot components such as orthogonality or parallelism, or the position of the reference frame. Errors from the robot can also arise due to stiffness, backlash, elasticity, and impact of temperature [16–18]. Camera intrinsic calibration is required to ensure that the images captured are of accurate dimensions and free of lens distortion, which would otherwise introduce errors in the measurement estimates that are fed back to the robot during operation. Hand-eye calibration ensures that the measurements made by the camera is converted to the reference used by the robot for measurement. The focus of this paper is on hand-eye calibration and its associated challenges to robotic vision system.

Hand-eye calibration is an absolute necessity for the accurate control of vision-based robotic systems. It enables the robot to obtain direct measurements of its environment via a camera to accurately perform its tasks. Hand-eye calibration estimates the pose (rotation and translation) of the robot’s end-effector (hand) with respect to the camera (eye) used for vision. This pose information is usually in the form of a homogeneous transformation matrix *X* between the end-effector frame and the camera frame and is usually formulated as $A_{a}^{b}X=XB_{a}^{b}$ [14], where $A_{a}^{b}$ and $B_{a}^{b}$ are the homogenous transformation matrices of the movement of the camera and the robot hand from frame *a* to *b* respectively.

**Fig 1:**
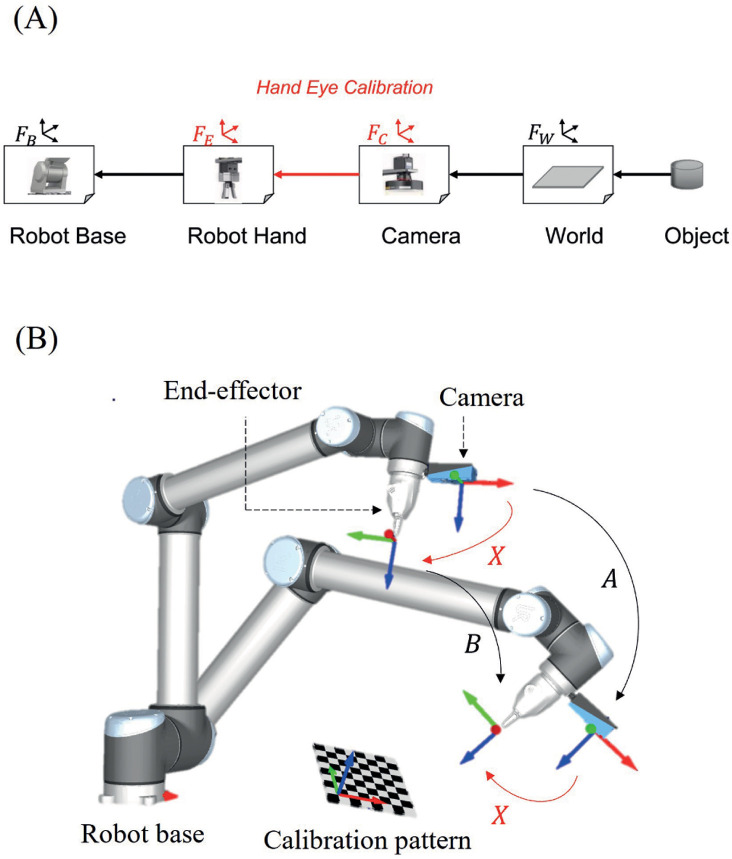
Component frames for vision-guided robot. (A) Frame relationships. (B) Hand-eye setup.

A homogeneous transformation matrix $H_{a}^{b}$ provides a convenient way of representing the relative rigid body transformation between two reference frames *a* and *b*. The matrix $H_{a}^{b}$ is composed of rotation $R_{a}^{b}$ of frame *b* with respect to frame *a*, and the translation $t_{a}^{b}$ of frame *b* with respect to frame *a*. Hence, a point *P*^*b*^ in frame *b* can be expressed relative to frame *a* as

$$P^{b}={H_{a}^{b}\;P}^{a}$$ (1A)

$$P^{b}=\left(\begin{array}{cc} R_{a}^{b} & t_{a}^{b}\\ 0^{T} & 1 \end{array}\right)\;P^{a}$$ (1B)

where $R_{a}^{b}$ is a 3 x 3 matrix, $t_{a}^{b}$ is a 3 x 1 vector and 0^*T*^ is a 1 x 3 zero vector. The properties of homogeneous transformation enable the realisation of arbitrary transformations by linking known transformations. In vision-guided robots, for example, the position of the surrounding objects relative to the robot base is usually required for appropriate robot control. As shown in Fig 1A, if the transformation $H_{e}^{b}$ from the robot base frame *F*_*B*_ to the robot hand frame *F*_*E*_, the transformation $H_{c}^{e}$ from the robot hand frame to the camera frame *F*_*C*_, and the transformation $H_{w}^{c}$ from the camera to the world frame *F*_*W*_ are known, then the position of the object in the world frame *P*^*w*^ can be obtained in the robot’s base frame *P*^*b*^ as $P^{b}=H_{e}^{b}\;H_{c}^{e}\;H_{w}^{c}\;P^{w}$. The transformation from the robot base to the robot hand $H_{e}^{b}$ can be directly obtained through the robot forward kinematics [19,20], while the transformation from the camera to the world $H_{w}^{c}$ can be obtained through the use of algorithms such as the Perspective-n-Point (P-n-P) [21], structure from motion [22] or other pose measurement algorithms [23], from a calibrated camera [24]. On the other hand, the process of obtaining the transformation from the robot hand to the camera $H_{c}^{e}$ referred to as the hand-eye transformation, forms what is known as the problem of hand-eye calibration [14]. The reliance of the hand-eye calibration on the robot [25–27] and camera information indicates the need for proper calibration of the robot and the camera [12,13,28].

Based on the work of Shiu and Ahmad [14], the hand-eye transformation can be obtained by solving the homogeneous transformation equation given by

AX=XB (2)

where, *A* and *B* are the homogeneous transformation matrices representation of the relative motions of the attached camera and the robot hand between two points, respectively, while *X* is the required homogeneous transformation between the robot hand and the camera as shown in Fig 1B. Eq (2) can be represented in a matrix form as

$$\left(\begin{array}{cc} R_{A} & {\vec{t}}_{A}\\ 0^{T} & 1 \end{array}\right)\left(\begin{array}{cc} R_{X} & {\vec{t}}_{X}\\ 0^{T} & 1 \end{array}\right)=\left(\begin{array}{cc} R_{X} & {\vec{t}}_{X}\\ 0^{T} & 1 \end{array}\right)\left(\begin{array}{cc} R_{B} & {\vec{t}}_{B}\\ 0^{T} & 1 \end{array}\right)$$ (3)

which can further be expanded to the rotation and translation parts as

$$R_{A}R_{X}=R_{X}\;R_{B}$$ (4A)

$$(R_{A}-I){\vec{t}}_{X}=R_{X}{\vec{t}}_{B}-{\vec{t}}_{A}$$ (4B)

where *R* is a 3 x 3 rotation matrix, $\vec{t}$ is a 3 x 1 translation vector and the subscripts *A*, *B* and *X* refers to the camera, robot and hand-eye pose respectively. Hence, the calibration operation involves obtaining sets of robot hand and camera poses. While the hand poses can easily be obtained from the robot forward kinematics using the joint encoder readings, the camera pose is usually estimated by observing a set of 3D points provided by a calibration object and their corresponding 2D images using the P-n-P algorithm [21,29]. While this formulation shows a more intuitive way to represent and solve the hand-eye problem, estimating the hand-eye transformation based on Eq (2) is not trivial. This is because the Special Euclidean SE(3) group structure of the homogeneous matrices must be preserved in the solution.

Several techniques have been proposed to solve the calibration problem, and they can broadly be classified as either the separated methods or the simultaneous methods. In the separated methods, the rotation parameter is first estimated from Eq (4A), then the translation parameter is estimated based on the estimated rotation using the linear Equation in Eq (4B). Shiu and Ahmed [14] and Tsai and Lenz [30] employed axis-angle parameterisation for the estimation of the rotation parameter while Chou and Kamel [31] and Park and Martin [32] used unit quaternions and Lie-algebra respectively to represent the rotation parameter.

Because the separated methods required estimating the translation parameter from the rotation parameter, errors from the rotation estimates are directly propagated to the translation estimates. Moreover, the separation of the rotation and translation parameters loses the inherent coupling between both parameters [33]. These arguments necessitated the need for simultaneous solutions to the hand-eye calibration problem. Chen [33] provided the first simultaneous solution to the hand-eye calibration problem. His method based on screw theory described the calibration problem as the rigid transformation between the screw axis of the hand and the camera. Zhao and Liu [34] extended the screw motion approach by representing the rotation with unit quaternions and formed a system of linear equations that were solved using Singular Value Decomposition (SVD). Daniilidis and Bayro-Corrochano [35] introduced the method of dual quaternion for solving the rotation and translation components simultaneously. Li et al. [15] applied Kronecker product in their approach but required additional orthogonalisation step to ensure a rotation matrix is realised.

In this study, we provide a systematic evaluation of how different factors (rotation noise, translation noise, rotational motion, translational motion) can impact the hand-eye calibration algorithms, through simulation with synthetic data and real experiment data. We also evaluate the computation time of each algorithm as a way to assess their relative complexities. Using six algorithms (Tsai and Lenz [30], Chou and Kamel [31], Park and Martin [32], Daniilidis and Bayro-Corrochano [35], Lu and Chou [36], and Li et. Al. [37]) as references, we comparatively show that the impact of those aforementioned factors does not follow a similar trend. The choice of these six algorithms is based on the performance evaluation from recent studies in comparison to other algorithms. From [38], the method from Park and Martin [32] showed the second-best accuracy based on Reconstruction Accuracy Error (RAE), while providing the best computation time based on the comparison of 4 hand-eye calibration algorithms. From [39] the methods form Daniilidis and Bayro-Corrochano [35], and Chou and Kamel [31] provided the best and second-best accuracies respectively in terms of relative pose error based on comparison of 10 hand-eye calibration algorithms. Based on the experimental results from [40], with increasing number of dataset, the method from Tsai and Lenz [30] provided the second best rotation accuracy, based on comparison with 5 other hand-eye calibration methods. The choice of the method from Li et.al. [37] was based on the fact that it provided the best accuracy when evaluated against two other methods that employed Kronecker product technique based on the relative rotation and translation errors. This was thus chosen as a candidate method for the evaluation of algorithms that utilise Kronecker product implementation. Furthermore, they also form the base idea on the development of most of the proposed hand-eye calibration algorithms, making them widely used for benchmarking [39,41] which gives them their popularity. These chosen algorithms also cover the different methods (separated and simultaneous) of generating solutions to the hand-eye calibration problem. In particular, the simultaneous methods are more resistant to rotation noise, whereas the separated methods are better at dealing with translation noise. Furthermore, while increasing the robot rotation motion span during calibration enhances the accuracy of the separated methods, it has a negative effect on the simultaneous methods. On the other hand, increasing the translation motion range improves the accuracy of simultaneous methods but degrades the accuracy of the separated methods. These findings suggest that those conditions should be considered when benchmarking algorithms or performing a calibration process for enhanced accuracy. The research contributions of this work are summarised as follows:

1. We provided insight on the effect of rotation and translation noise acting in isolation, on the estimated hand-eye calibration parameters. We went further to use the different algorithms to quantitatively assess the impacts. Insights from this assessment would spur further studies on how this can be used to an advantage in minimising estimation errors. For instance, restricting the robot motion to either rotation or translation while performing hand-eye calibration.
  Previous studies have mainly considered pose noise which is a combination of both rotation and translation noise.
2. We provided insight on the effect of the range of robot motion used during the hand-eye calibration operation. This has rarely been a criterion for benchmarking hand-eye calibration algorithms. Our results show that the range of motions (rotation and translation) used during the calibration operation has a significant impact on the accuracy of the hand-eye calibration parameter. Our results also suggest that the rotation motions have different effect on the accuracy from the translation motions. We reckon this would be an application constraint, for instance in applications where size and mass are a premium such as in space applications. As such, the result from this comparison would be helpful as a guide in selection of algorithm or inform future benchmarking of proposed algorithms.

## Materials and methods

### Nomenclature

*R*: 3 x 3 rotation matrix

$\vec{t}$: 3 x 1 translation vector

$[\vec{v}]{}_{\times }$: Skew of vector $\vec{v}$ such that $[\vec{v}]{}_{\times }=\left(\begin{array}{ccc} 0 & {-v}_{3} & v_{2}\\ v_{3} & 0 & {-v}_{1}\\ -v_{2} & v_{1} & 0 \end{array}\right)$

$q(q_{0},\vec{q})$: Unit rotation quaternion made up of a scalar part *q*_0_ and a vector part $\vec{q}$

*q*^±^: Representation of a quaternion to aid matrix multiplication such that

$$q^{\pm }=(\begin{array}{cc} q_{0} & -{\vec{q}}^{T}\\ \vec{q} & q_{0}I\mp [\vec{q}]{}_{\times } \end{array}),$$

where *I* is the identity matrix

*q*′: The dual of a quaternion

*sign*(*x*): Defines the sign of a value such that

$$sign(x)=\left\{ \begin{array}{l} -1,\;x<0\\ \;1,\;x\geq 0 \end{array}\right.$$

*vec*(*A*): Vectorization of matrix *A*

⊗: Kronecker product

### Hand-eye calibration methods

The hand-eye calibration methods can be categorised based on the representation of the rotation parameter such as angle-axis, quaternions, dual-quaternions, Lie group, etc. They can also be described based on the parameter estimation procedure as a separable solution or simultaneous solution. In this section, we provide an overview of the six hand-eye calibration algorithms which will be evaluated in this study from the perspective of easy-of-implementation and reproducibility of the presented algorithms. These six algorithms are Methods of Tsai and Lenz [30], Chou and Kamel [31], Park and Martin [32], which are the separated methods and Methods of Daniilidis and Bayro-Corrochano [35], Lu and Chou [36], and Li et.al. [37], which are the simultaneous methods. These methods have been chosen for this study because they not only form the foundations of many hand-eye calibration algorithms but are frequently used for benchmarking newer hand-eye calibration methods. For a complete overview of these algorithms, the readers are encouraged to see the accompanying references.

### Method of Tsai and Lenz (1989) [30]

The Method of Tsai and Lenz [30] (hereinafter termed Method Tsai) provides a separable solution to the hand-eye calibration problem using the angle-axis representation of the rotation parameter *R*_*X*_ given by

$$R_{X}=Rot({\vec{n}}_{X},\theta_{X})$$ (5)

where ${\vec{n}}_{X}$ is the axis of rotation and *θ*_*X*_ is the angle of rotation. Using this method, the rotation axis and angle can be computed by

$${\left[{\vec{n}}_{A}+{\vec{n}}_{B}\right]}_{\times }{\vec{n}\prime }_{X}={\vec{n}}_{A}-{\vec{n}}_{B}{\vec{n}}_{X}=\frac{2{\vec{n}\prime }_{X}}{\sqrt{1+∥{\vec{n}\prime }_{X}{∥}^{2}}}$$ (6A)

$$\theta_{X}=2{tan}^{-1}(∥{\vec{n}\prime }_{X}∥)$$ (6B)

where ${\vec{n}}_{A}$ and ${\vec{n}}_{B}$ are the axes of rotation in the camera and hand frames, respectively.

The rotation matrix *R*_*X*_ can be obtained by

$$R_{X}=\left(1-\frac{∥{\vec{n}}_{X}{∥}^{2}}{2}\right)\cdot I+0.5\left[{\vec{n}}_{X}∙{\vec{n}}_{X}^{T}+(\sqrt{4-∥{\vec{n}}_{X}{∥}^{2}}){\left[{\vec{n}}_{X}\right]}_{\times }\right]$$ (7)

The motivation of this approach is to provide a solution by solving a fixed linear system of equations, as the earlier approach [14] required an increasing number of equations for each additional robot motion used in the calibration.

### Method of Chou and Kamel (1988)

Chou and Kamel [31] (hereinafter termed Method Chou) represented the rotation with unit quaternions and formulated the calibration Eq (6A) as

$$q_{A}q_{X}=q_{X}q_{B}$$ (8)

where $q_{A}(a_{0},\vec{a}),\;q_{B}(b_{0},\vec{b})$ and $q_{X}(x_{0},\vec{x})$ are the unit quaternions representing rotations in the camera, robot, and hand frames, respectively. Using quaternion matrix multiplication, Eq (8) can be written as

$$(q_{A}^{+}-q_{B}^{-})q_{X}=0$$ (9)

or

$$Gq_{X}=0$$ (10)

where

$$G=\left(\begin{array}{cc} a_{0}-b_{0} & -{\left(\vec{a}-\vec{b}\right)}^{T}\\ \vec{a}-\vec{b} & (a_{0}-b_{0})I+{\left[\vec{a}\right]}_{\times }+{\left[\vec{b}\right]}_{\times } \end{array}\right)$$

The solution to the hand-eye transformation can easily be obtained from Eq (10) via SVD.

#### Method of Park and Martin (1994) [32]

Park and Martin [32] (hereinafter termed Method Park) formulated a computationally more efficient and linearised method by parameterising the rotation with Lie-group. This provides a logarithmic mapping from the *SO*(3) group to the corresponding *so*(3) Lie algebra, where *SO*(3) and *so*(3) represent the special orthogonal group matrices of size 3 x 3 and the corresponding Lie algebra matrix of size 3 x 1, respectively. Given that

$$log(R)=\frac{\theta }{2sin\theta }(R-R^{T})$$ (11)

such that *θ* satisfies 1+2*cosθ* = *tr*(*R*), where *tr*(*R*) is the trace of *R*. The rotational part of the calibration Eq (4A) can be represented by its logarithmic mapping as

$$R_{X}\beta_{i}=\alpha_{i}$$ (12)

where *α* and *β* are *log*(*R*_*A*_) and *log*(*R*_*B*_), respectively. *R*_*X*_ can be obtained by least-square minimisation such that

$$R_{X}={\left(M^{T}M\right)}^{-\frac{1}{2}}M^{T}$$ (13A)

$$M=\sum_{i=1}^{N}\beta_{i}\alpha_{i}^{T}$$ (13B)

where *N* is the number of data points.

While this method is computationally efficient and does well in the presence of noise, the computation of *log*(*R*_*A*_) and *log*(*R*_*B*_) imposes a restriction that *R*_*A*_ and *R*_*B*_ must be rigid transformations, otherwise, it becomes impossible to compute their logarithms.

#### Method of Daniilidis and Bayro-Corrochano (1996) [35]

Daniilidis and Bayro-Corrochano [35] (hereinafter termed Method Daniilidis) provided an algebraic interpretation of the screw motion approach to hand-eye calibration [33] using dual quaternion representation. For a unit quaternion $q(q_{0},\vec{q})$ representing the rotation in a rigid body transformation, its dual $q\prime ({q\prime }_{0},\vec{q}\prime )$ is given as

$$q\prime =\frac{1}{2}tq$$ (14)

where *t* is the translation component of the transformation. Using only the vector part of the dual quaternion representation of the camera and robot transformation, i.e, $a_{i}(0,{\vec{a}}_{i}),\;a\prime_{i}(0,\vec{a}\prime_{i})$ and $b_{i}(0,{\vec{b}}_{i}),\;b\prime_{i}(0,\vec{b}\prime_{i})$, respectively, Eq (2) can be formulated as

$$\left(\begin{array}{cccc} \vec{a}-\vec{b} & {\left[\vec{a}+\vec{b}\right]}_{\times } & 0_{3\times 1} & 0_{3\times 3}\\ {\vec{a}}^{\prime }-{\vec{b}}^{\prime } & {\left[{\vec{a}}^{\prime }+{\vec{b}}^{\prime }\right]}_{\times } & \vec{a}-\vec{b} & {\left[\vec{a}+\vec{b}\right]}_{\times }0_{3\times 1}0_{3\times 3} \end{array}\right)\left(\begin{array}{c} q_{x}\\ q_{x}^{\prime } \end{array}\right)=0$$ (15)

This matrix has two singular vectors ${\vec{u}}_{1}^{T}=({\vec{v}}_{1}^{T},{\vec{w}}_{1}^{T})$ and ${\vec{u}}_{2}^{T}=({\vec{v}}_{2}^{T},{\vec{w}}_{2}^{T})$ that span the null-space and hence satisfy the equation

$$\left(\begin{array}{c} q_{x}\\ q_{x}^{\prime } \end{array}\right)=\lambda_{1}\left(\begin{array}{c} {\vec{v}}_{1}\\ {\vec{w}}_{1} \end{array}\right)+\lambda_{2}\left(\begin{array}{c} {\vec{v}}_{2}\\ {\vec{w}}_{2} \end{array}\right)$$ (16)

To ensure the result is a unit dual quaternion, Eq (16) must be solved together with the constraints given by

$$q_{x}^{T}q_{x}=1$$ (17A)

$$q_{x}^{T}{q\prime }_{x}=0$$ (17B)

This leads to the formation of two quadratic equations in *λ*_1_ and *λ*_2_ from which $\left(q_{x},q_{x}^{\prime }\right)$ can be determined.

#### Method of Lu and Chou (1995) [36]

Lu and Chou [36] (hereinafter termed Method Lu) proposed a simultaneous solution by formulating a linear system of equations using quaternion given by

$$\left(\begin{array}{cc} P & Q\\ Q & 0 \end{array}\right)\left(\begin{array}{c} q_{x}\\ t_{x}^{\prime } \end{array}\right)=0$$ (18)

where $q_{x}(q_{0_{x}},{\vec{q}}_{x})$ is the unit quaternion representation of the rotation and the translation component *t*_*x*_ is given by

$$t\prime_{x}=E^{T}t$$ (19)

where $E=(-{\vec{q}}_{x}q_{0_{x}}I+{\left[{\vec{q}}_{x}\right]}_{\times })$ while *P* and *Q* are given by

$$P=q_{b}^{-}(t_{b}^{+}-t_{a}^{-})$$ (20A)

$$Q=q_{b}^{+}-q_{a}^{-}$$ (20B)

where *q*_*a*_ and *q*_*b*_ are the quaternion representation of the rotation of the camera frame and robot hand frames respectively, *t*_*a*_ and *t*_*b*_ are the quaternion representation of the translation of the camera frame and robot hand frames, respectively. This system must be solved with the constraint given by

$$q_{x}^{T}q_{x}=1$$ (21A)

$$q_{x}^{T}t\prime_{x}=0$$ (21B)

#### Method of Li et. Al. (2018) [37]

To simultaneously solve for the rotation and translation components Li et. Al., [37] (hereinafter termed Method Li) described the calibration given in Eq (4) using the relationship between matrix vectorisation and the Kronecker product. The vectorisation of the product of matrices *A*, *B* and *C* can be written as

$$vec(ABC)=(C^{T}⊗A)vec(B)$$ (22)

Eq (4) can thus be written as

$$\left(\begin{array}{cc} I_{9}-(R_{B}⊗R_{A}) & 0_{9\times 3}\\ t_{B}^{T}⊗{\left[t_{A}\right]}_{\times } & {\left[t_{A}\right]}_{\times }(I_{3}-R_{A}) \end{array}\right)\left(\begin{array}{c} vec({\hat{R}}_{X})\\ {\hat{t}}_{X} \end{array}\right)=\left(\begin{array}{c} 0_{9\times 1}\\ t_{A} \end{array}\right)$$ (23)

Eq (23) is thus a linear system that can be solved by the well-known least-squares method. To ensure that the recovered rotation meets the constraints that its determinant is 1, a proportionality constant *ω* can be calculated as

$$\omega =sign\left(det({\hat{R}}_{X})\right)det{\left({\hat{R}}_{X}\right)}^{-\frac{1}{3}}$$ (24)

The recovered rotation and translation can thus be given as

$$R_{X}=\omega {\hat{R}}_{X}$$ (25A)

$$t_{X}=\omega {\hat{t}}_{X}$$ (25B)

### Performance evaluation metrics

The following evaluation metrics were used to comparatively evaluate the performance of the different algorithms, each of which has its property and usefulness.

#### Relative transformation error

The relative transformation error *E*_*rn*_ is unitless and is derived from Eq (2). It evaluates how close the rigid transformation on the left side of the equation is to the right side of the equation based on the estimated hand-eye transformation *X*. The relative transformation error is given by

$$E_{rn}=\frac{1}{N}\sqrt{\sum_{i=1}^{N}∥A_{i}X-XB_{i}{∥}^{2}}$$ (26)

#### Rotation error

Two forms of rotation errors are utilised for this evaluation: the relative rotation error *E*_*R*_, and the mean absolute rotation error $E_{R_{x}}$ These are given by

$$E_{R}=\frac{1}{N}\sum_{i=1}^{N}angle[{\left(R_{X}R_{B_{i}}\right)}^{T}(R_{A_{i}}R_{X})]$$ (27A)

$$E_{R_{x}}=\frac{1}{N}\sum_{i=1}^{N}angle({\hat{R}}_{X_{i}}^{-1}R_{X})$$ (27B)

where ${\hat{R}}_{X_{i}}$ is the rotation estimate value of the hand-eye transformation during simulation. The relative rotation error, Eq (27A), is suitable for evaluation with real data where the ground-truth hand-eye transformation is not available. For simulation study where the ground-truth data is available, then, it becomes more useful to use the mean absolute rotation error $E_{R_{x}}$ given in Eq (27B).

#### Translation error

Following the rotation errors, the relative translation error *E*_*T*_ and the mean absolute translation error $E_{T_{x}}$ are used in this study for real data and simulation studies, respectively, and they are given by

$$E_{T}=\frac{1}{N}\sum_{i=1}^{N}∥(R_{A_{i}}t_{X})-t_{X}-(R_{X}t_{B_{i}})+t_{A_{i}}∥$$ (28A)

$$E_{T_{x}}=\frac{1}{N}\sum_{i=1}^{N}∥t_{X}-{\hat{t}}_{X}∥$$ (28B)

## Materials and methods

### Real dataset collection

**Fig 2:**
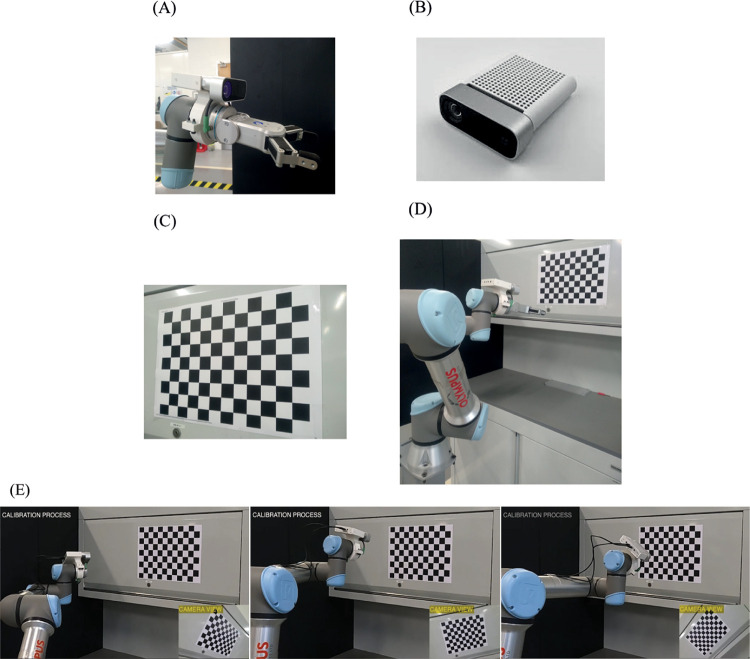
Hand-eye calibration setup. (A) Camera-End-Effector setup. (B) Camera. (C) Calibration pattern. (D) Experiment setup (E) Poses of robot and camera view representing camera pose during calibration process.

For this experiment, we used a UR5e robot arm rigidly mounted on the floor to provide the robot pose data and a Microsoft AzureKinect camera secured to the last link of the robot for the image acquisition which is used to compute the camera poses. A 32mm, 11 by 8 checkerboard pattern was used as the calibration target. During the experiment, the robot arm was moved to a range of positions with the calibration pattern still in the view of the camera for image acquisition. The control of the robot motion was achieved through an interface with RoboDK running a script written in Python. This ensured a high-level interaction with the robot which makes for easy implementation. During the experiments, software checks was implemented to detect and avoid configuration changes in the robot. Also, the motion of the robot was restricted such that the rotational angle was below 180 degs. This ensures that the issue of singularity was avoided during the computation of the hand-eye calibration parameters which occurs close to or at this threshold [14,30]. The robot poses were obtained directly from the robot pendant while the camera poses were estimated using the P-n-P algorithm from the OpenCV library [42]. The setup is shown in Fig 2. A demo video of the calibration operation can be found in S1 Video in S1 Appendix. For the evaluation of hand-eye calibration algorithms, a total of 101 robot poses and images of size 1280 x 720 pixels were acquired.

**Fig 3:**
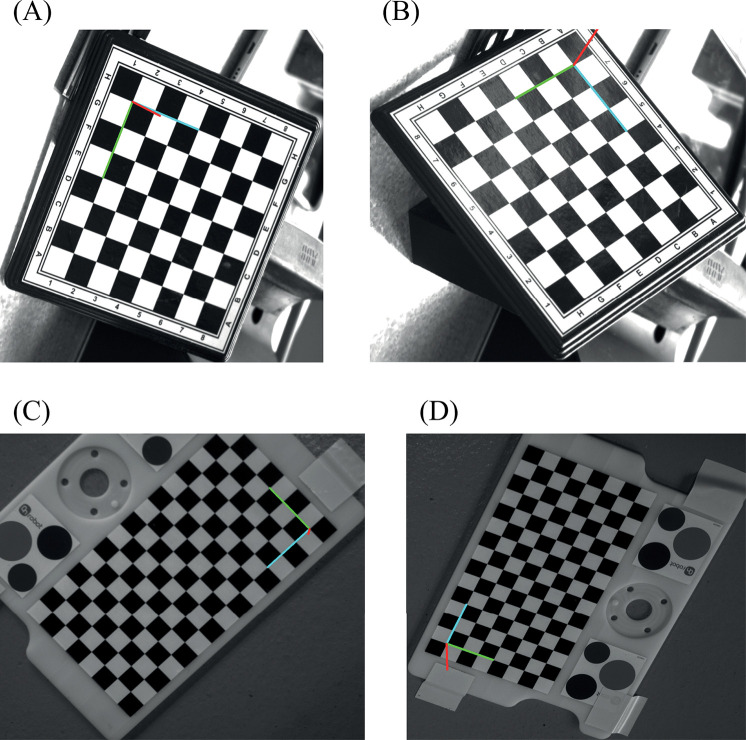
Change in position of origin (red, green, and blue axes) with change in orientation of target for an odd number of rows and columns. (A) Origin at position 1H. (B) Origin at position 7A. (C) Origin at the bottom right. (D) Origin at the bottom right after rotation.

During the camera pose computation, we noted that the position of the origin as detected by the P-n-P algorithm from the OpenCV library was sensitive to the orientation of the calibration pattern in the image when both the rows and columns used are of even or odd number as shown in Fig 3. Fig 3 shows the origin of the target reprojected on the image after pose estimation with the OpenCV P-n-P library. In Fig 3A, the origin is located at position 1H on the chessboard. However, when the chessboard is rotated sufficiently as in Fig 3B, the origin location changes to position 7A. This leads to a loss in the actual computed rotation.

This change in the origin affected the actual camera pose estimate. Using odd and even numbers of rows and columns in the calibration pattern, however, forced the algorithm to be consistent in the position of the origin for every pose acquisition as shown in Fig 3C and 3D.

### Simulation dataset generation

Using real dataset ensures that the overall dynamics and uncertainties in the system are captured. However, because it is impossible to get the ground truth data for the hand-eye transformation, it becomes impossible to make an absolute evaluation of the performances of the different algorithms based on their true rotation and translation estimates. As such, synthetically generated data becomes useful for the study. This also allows for a quick, easy, and in-depth study of various scenarios for hand-eye calibration where the parameters can be controlled.

For the simulation study, random ground truth data was chosen for *X* in terms of its translation vector *t*_*X*_, *t*_*Y*_, *t*_*Z*_ and rotation (Euler) angles *R*_*X*_, *R*_*Y*_, *R*_*Z*_. These values were then converted to the required homogeneous transformation matrix. The same procedure was followed for generating the various robot pose data *B* and the position of the world coordinate *W*. The camera pose *A*_*i*_ for each robot pose *B*_*i*_ was then calculated using *A*_*i*_ = *WB*_*i*_*X*^−1^.

### Pose error generation

During hand-eye calibration of an actual robot-camera system, the two sources of errors are from the robot and the camera. Because the data are in the form of poses, these errors must also pose errors, which can be interpreted as the transformation *δ*_*B*_ that moves the robot hand from their measured position $\hat{B}$ to their actual position *B*, such that $\delta_{B}={\hat{B}}^{-1}B$. For the camera motion, the error *δ*_*A*_ is the transformation that moves the camera from its expected position *A* to its measured position $\hat{A}$, such that $\delta_{A}=A^{-1}\hat{A}$. During the calibration operation, the robot poses are obtained from the robot forward kinematics, which is generally available from the robot control interface or pendant. As such only the measured pose of the robot is available. However, the robot pose error can be modelled by reflecting it in the camera pose measurements. This error in camera pose $\delta_{A_{B}}$ from the reflection of robot pose error *δ*_*B*_ can be expressed as $\delta_{A_{B}}=X\delta_{B}X^{-1}$. Hence, during the simulation study, defining total simulation pose error $\delta_{e}=\delta_{A_{B}}\delta_{A}$, then the following equation, $A\delta_{A_{B}}\delta_{A}X=XB$ can be used to estimate the hand-eye transformation *X* in the presence of pose error in the robot and camera, with *A* and *B* are the ideal relative camera and robot poses respectively.

## Results and discussions

### Simulation study

In our simulation study, we generated the robot and camera pose data as described in the previous section. The robot poses were based on a uniform distribution, such that the Euler rotations [*θ*_*X*_, *θ*_*Y*_, *θ*_*Z*_] ∈ *U*(−180,180) (deg) and translation [*t*_*X*_, *t*_*Y*_, *t*_*Z*_] ∈ *U*(−1000,1000) (mm). To study the sensitivity of various algorithms to noise in the robot and camera pose measurements, we generated random noise poses with Gaussian distribution in the rotation based on the Euler angles (deg) and translation (mm) with zero mean *μ* and varied the standard deviation *σ*. The converted homogeneous transformation noise *δ*_*e*_ was then added to the pose data. We conducted the simulation by executing 100 simulation runs at each estimation step, sampling the noise from its Gaussian distribution. The choice of 100 simulation runs follows from [43,44] and provides a trade-off between total simulation time and statistical significance that arise from a large number of experiments. All simulations are based on a Python implementation of the algorithms and evaluation techniques running on a Windows PC with Intel i7-2.7GHz CPU and 16GB of RAM.

#### Effect of number of robot motions

**Fig 4:**
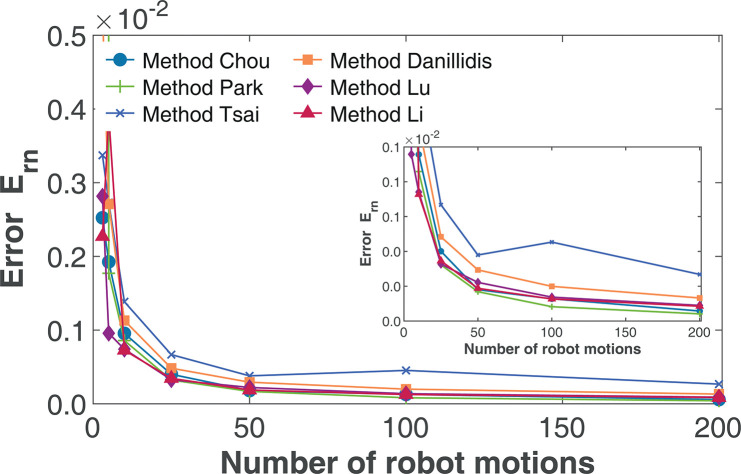
Effect of the number of robot motions on relative transformation error *E*_*rn*_(*σ*_*r*_ = *σ*_*t*_ = 0.5). The inset shows the zoomed in accuracy level between *E*_*rn*_ of 0 and 0.001.

We evaluated the performance of the various algorithms based on the number of robot poses used for the calibration. For this study, we performed a total of 100 simulation runs while keeping the standard deviation of the rotation *σ*_*r*_ and translation *σ*_*t*_ noise fixed at 0.5 and 1, respectively. Given that the minimum number of robot poses for a valid computation of the hand-eye parameter is 3 [14], we varied the number of robot poses from 3 to 200. Fig 4 shows the result of the simulation using the relative transform error evaluation *E*_*rn*_.

From Fig 4, we can observe that for an increasing number of robot motions used in the calibration operation, all the evaluated algorithms show an increase in the accuracy given by the relative transformation error. Furthermore, the result makes it evident that as the number of robot motions used increases, the gain in estimation accuracy becomes minimal. For the number of 3 to 50 robot motions, the result shows a significant drop in the error. However, after 50 robot motions, only a minimal decrease in the error is observed. For other simulation studies, the number of 100 robot motions will be used as it is evident that all the algorithms perform better at a higher number of robot motions.

#### Effect of rotation noise

**Fig 5:**
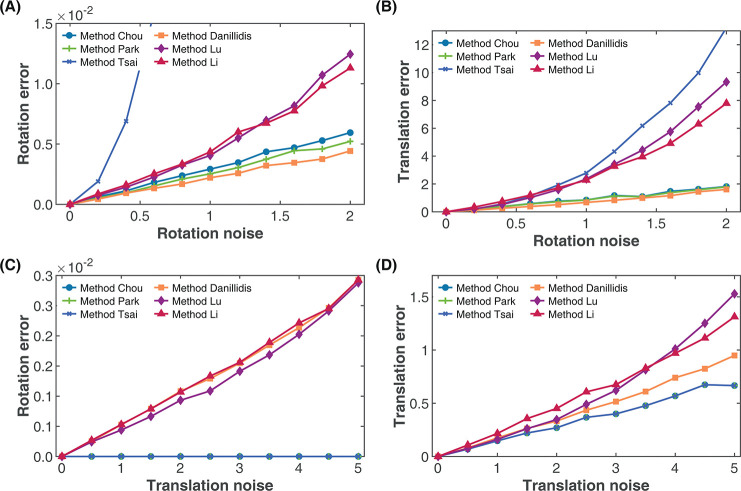
Effect of rotation and translation noise on rotation and translation calibration accuracy. (A) Effect of rotation noise on rotation accuracy. (B) Effect of rotation noise on translation accuracy. (C) Effect of translation noise on rotation accuracy. (D) Effect of translation noise on translation accuracy.

For this simulation study, we aim to observe how the rotation and translation estimates are affected by noise from the rotation component alone. We set the number of robot motions to 100 and varied the standard deviation of the rotation noise *σ*_*r*_ from 0 to 2 without any translation noise (*σ*_*t*_ = 0). For each noise sampling, we performed a total of 100 simulations and computed the mean absolute rotation and translation errors. Fig 5A and 5B show the result of the simulation, where we observe the accuracy of the rotation estimates based on absolute rotation error decrease with increasing rotation noise as expected. However, from Fig 5A, the rotation estimates based on Method Daniilidis showed the best performance with increasing rotation noise. While the performance of Method Chou and Method Park are not far off from Method Daniilidis, that of Methods Li and Lu which were similar became significantly worse as the rotation noise increases. The performance of Method Tsai on the other hand appeared to be very sensitive to rotation noise and provided large rotation error even at lower rotation noise.

Considering the effect of the rotation noise on the estimates of the translation parameter, Fig 5B suggests that the translation estimates provided by Method Daniilidis, Method Chou and Method Park based on absolute translation error also remained more robust to translation noise than the other methods in the absence of rotation noise, with Method Daniilidis showing slightly better translation estimates. The translation estimates of Method Li, Method Lu and Method Tsai progressively became worse as the rotation noise increased, with the latter providing the best translation estimate of the three at low rotation noise (*σ*_*r*_<0.5). At higher rotation noise however, the translation estimates of Methods Tsai became the worst. Since the only noise present is from the rotation component, the errors in the translation components are propagated from the rotation components as argued in numerous literature on hand-eye calibration as a need for simultaneous solution [35,45,46]. However, it becomes apparent that for simultaneous methods, as seen in the performance of Method Daniilidis and Method Lu (Fig 5B), errors can be induced in the translational component as well in the presence of rotation error.

#### Effect of translation noise

To study the effect of translation noise on the calibration accuracy, we used 100 motions of the robot with no rotation noise (*σ*_*r*_ = 0) while varying the standard deviation of the translation noise *σ*_*t*_ from 0 to 5. We then performed 100 simulation runs and calculated the mean absolute rotation and translation errors as shown in Fig 5C and 5D. Fig 5C shows the accuracy of the rotation estimate in the presence of translation noise. Based on the observed result, the separated methods (Method Chou, Method Park and Method Tsai) show robustness against the translation noise from the robot motion. This is expected as the rotation parameter is computed without the translation component.

For the methods with the simultaneous solutions (Method Danillidis, Method Lu and Method Li), the result shows increasing error in the estimated rotation with an increase in the translation noise. Amongst the three simultaneous methods evaluated, Method Daniilidis and Method Li showed similar performance, however, Method Lu provided the best rotation estimates under translation noise as the only source of the noise. From the point of view of the translation estimate as seen in Fig 5D, while all the separated methods had similar translation accuracy, as the translation noise increases from a variance of 0, the accuracy level of the simultaneous solution methods became progressively worse compared to the separated methods, with Method Daniilidis showing the best performance of the three simultaneous methods. The superior performance of the separate methods compared to the simultaneous methods is attributed to the estimation of the translation parameter with least square on a linear system rather than the non-linear system provided by the simultaneous methods.

#### Combined effect of noise on the rotation and translation estimates

In the previous section, we looked at the effect of the noise from the rotation and translation components on the estimated rotation and translation parameters, where each noise source acted alone. The results suggest that the simultaneous method of Method Daniilidis and the separated methods of Method Chou and Method Park are more robust to noise in rotation and translation with Method Daniilidis slightly better. Method Tsai on the other hand appeared to be extremely sensitive to high rotation noise levels while Methods Li and Lu showed roughly similar performance. These results give an idea of the sensitivity of the different algorithms to noise from each of the components, however, in reality the algorithms would have to handle the combined noise from both sources, which is not a linear function. To evaluate the sensitivity of the different algorithms to the noise from the rotation and translation components acting together, we simultaneously increased both the rotation and translation noise variance with *σ*_*r*_ = (0,2) and *σ*_*t*_ = (0,5), respectively.

**Fig 6:**
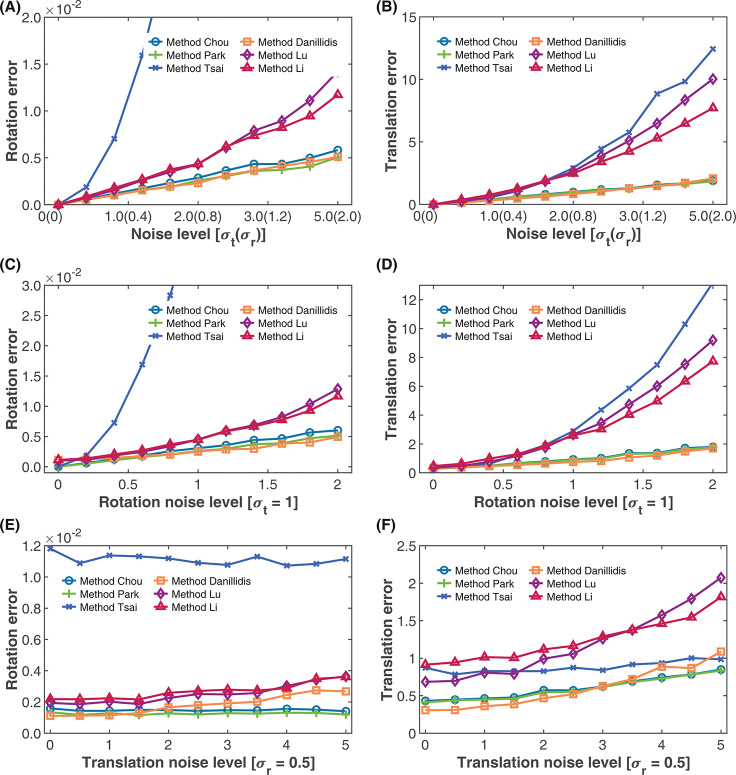
Effect of combined rotation and translation noise on calibration accuracy. (A) Effect of increasing rotation and translation noise on rotation accuracy. (B) Effect of increasing rotation and translation noise on translation accuracy. (C) Effect of rotation noise on rotation accuracy with fixed translation noise *σ*_*t*_. (D) Effect of rotation noise on translation accuracy with fixed translation noise *σ*_*t*_. (E) Effect of translation noise on rotation accuracy with fixed rotation noise *σ*_*r*_ (F) Effect of translation noise on translation accuracy with fixed rotation noise *σ*_*r*_.

The result of this evaluation based on the average of 100 simulation runs is shown in Fig 6A and 6B. From Fig 6A, with increasing rotation and translation noise, Method Daniilidis and Method Park showed roughly similar and better performance than the others with Method Chou only slightly worse. On the other hand, Method Li and Method Lu again showed similar performance, with Method Lu slightly better at lower joint rotation and translation noise levels (*σ*_*r*_<1.4, *σ*_*t*_<3.5), while Method Lu appear slightly better at higher noise levels. Method Tsai, as in the previous evaluations showed large rotation errors as the noise levels increased. From Fig 6B, Methods Daniilidis, Park and Chou again show the best performance for translation estimates. However, Method Daniilidis was slightly better at lower rotation and translation levels (*σ*_*r*_<1, *σ*_*t*_<2.5). With increasing rotation and translation noise levels, the estimated translation errors of Methods Tsai, Lu and Li increases progressively. Of these three methods, Method Tsai proved the best at lower noise levels (*σ*_*r*_<0.8, *σ*_*t*_<2) but the worst at higher noise levels. Method Li on the other hand showed the worst performance at lower noise levels (*σ*_*r*_<0.8, *σ*_*t*_<2), but the best at higher noise levels.

**Fig 7:**
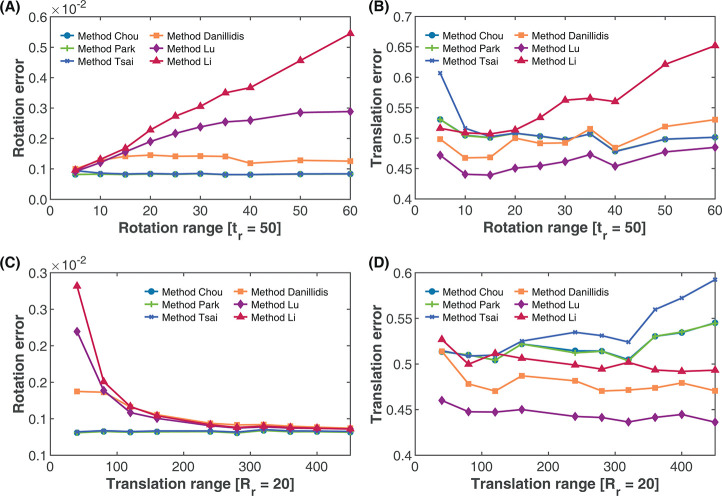
Effect of rotation and translation motion on estimation accuracy. (A) Effect of rotation motion on rotation accuracy with fixed translation range. (B) Effect of rotation motion on translation accuracy with fixed translation range. (C) Effect of translation motion on rotation accuracy with fixed rotation range. (D) Effect of translation motion on translation accuracy with fixed rotation range.

To get more dynamic insights into the performance, we set the noise variance of the rotation, or the translation components fixed while varying the other. The results based on the average of 100 simulation runs are shown in Fig 6C to 6F. In Fig 7C and 7D, we kept the translation noise variance fixed at *σ*_*t*_ = 1 and varied the rotation variance from *σ*_*r*_ = 0 to *σ*_*r*_ = 2, while in Fig 6E and 6F, we kept the rotation noise variance fixed at *σ*_*r*_ = 0.5 and varied the translation variance from *σ*_*t*_ = 0 to *σ*_*t*_ = 5.

From Fig 6C, it can be observed that for the same translation noise, all the separated methods–Method Chou, Method Park and Method Tsai—showed better performance at lower rotation noise (*σ*_*r*_<0.6) than the simultaneous methods. However, as the rotation noise increased, the performance of Method Daniilidis became better than all the separated methods. For the range of noise levels evaluated, Method Daniilidis, Method Chou and Method Park consistently provided better rotation estimates than the other methods. Methods Lu and Li again showed similar performance but with Method Lu slightly performing better at lower rotation noise (*σ*_*r*_<1) than Method Li, while Method Tsai showed a good performance only at very low rotation noise levels (*σ*_*r*_<0.125). For the translation estimates (Fig 6D) all the separated methods again performed better only at rotation noise variance below 0.25. At higher rotation noise levels, the performance of Method Daniilidis becomes better than all the separated methods. While the translation errors of Methods Chou and Park which were similar were only marginally higher than Method Daniilidis at higher rotation noise, the translation errors Method Tsai rose above those of Method Lu and Method Li at rotation noise variance above 0.75. With increasing the translation noise at fixed rotation noise, it can be observed from Fig 6E that the rotation estimates of the separated methods remained relatively stable with Methods Chou and Park showing low rotation error while Method Tsai showed high rotation error. The stable rotation estimate with increasing translation noise at fixed rotation noise is expected as the rotation is estimated without the translation parameter. Hence the error in the separated methods is due only to the rotation error. However, Method Daniilidis showed better rotation estimates than the separated methods at low translation noise (*σ*_*r*_<1) after which its performance degraded further with increasing translation noise. From Fig 6F, it can be noticed that while all the methods showed increase in translation error with increasing translation noise, the increase in translation error is more pronounced for the separated methods. At high translation noise levels (*σ*_*t*_>4.75) the performance of all the simultaneous methods became worse than the separated methods.

#### Effect of robot motion range

Here, we aim to observe how the range of motion of the robot in rotation and translation affects the calibration accuracy. For this simulation, first, we restricted the robot translation to a range *t*_*f*_ = 50 mm and varied the rotation around each of the axes from a range of *R*_*r*_ = 5 deg to *R*_*r*_ = 60 deg. Here the range *x*_*r*_ of *x* is defined as

$$x_{r}=U(0.9x,x)$$ (29)

Secondly, we restricted the rotation of the robot motion to a range of *R*_*f*_ = 20 deg while varying the translation motion *t*_*r*_ between 40 mm to 450 mm. The translation range *t*_*r*_ is calculated based on the norm of the translation as

$$t_{r}=|t|,$$ (30)

while the rotation range *R*_*r*_ is calculated as

$$R_{r}=Rot(R)$$ (31)

where *R* is the rotation matrix.

This simulation was done over a total of 100 robot motions with the standard deviation of the rotation and translation noise set to *σ*_*r*_ = 0.1 and *σ*_*t*_ = 0.5, respectively. We then calculated the relative rotation and translation errors, and the results are shown in Fig 7.

As seen in Fig 7A, increasing the range of rotation of the robot during hand-eye calibration at a constant translation rate has a marginal effect on the rotation estimates for the separated methods. This increment appears to be more pronounced in Method Tsai from a very low rotation (below 10 deg) than Methods Chou and Park where the accuracy improvement appears minimal. For the separated methods, however, the accuracy of the rotation estimates decreases when the rotation range is increased at a constant translation range. A similar trend is observed with the translation estimates based on the rotation span in Fig 7B, which shows a decreasing translation error with increasing rotation motion for the separated methods, while the translation error of the simultaneous methods increased with increasing rotation motion span.

In terms of the effect of the translation motion span on the estimation accuracy, Fig 7C shows a significant reduction in the rotation error of the simultaneous methods, while the rotation error of the separated methods increased marginally. Furthermore, from Fig 7D, increasing the translation motion span resulted in the increase in the accuracy of the simultaneous methods while exhibiting a decrease in the accuracy of the separated methods.

#### Simulation time

Here, we are interested in the execution time of the algorithms in performing the hand-eye calibration. We have considered 20 and 100 robot motions and executed 100 simulation runs for each algorithm. The execution time was averaged over the simulation runs. This evaluation is based on a Python implementation of the algorithms running on a PC with Intel i7-2.7GHz CPU and 16GB of RAM. The result is shown in Table 1.

**Table 1:** Comparison of algorithm execution time for 20 and 100 robot motions.

Algorithms	Execution time (seconds)	
	20 robot motions	100 robot motions
Method Chou	0.084	0.444
Method Park	0.062	0.301
Method Tsai	0.079	0.399
Method Daniilidis	0.119	0.558
Method Lu	0.107	0.504
Method Li	0.096	0.887

The result from Table 1 suggests that Method Daniilidis is the most computationally expensive in comparison with the other methods for a lower number of robot motions. However, as the number of robot motions increases, the execution time of Method Li increases and is the most computationally expensive compared to the other methods. The large computational time for Method Daniilidis at a number of low robot motions can be attributed to the need to solve a dual variable polynomial. However, Method Li employs Kronecker product which has a quadratic complexity *O*(*n*^2^), as such its processing time increases progressively with the amount of data. Method Park appeared to be the most computationally efficient method in both scenarios. Interestingly, all the three separated methods are shown to be more computationally efficient than the simultaneous methods.

### Experimental evaluation with UR5e robot

**Fig 8:**
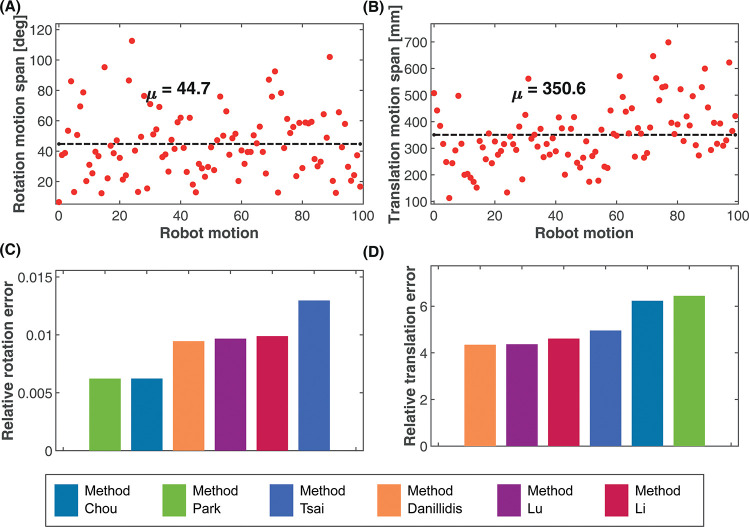
Random rotation and translation motion of the robot during data collection. (A) Rotation motion span, mean, *μ* = 44.7 deg. (B) Translation motion span, mean *μ* = 350.6 mm. (C) Relative rotation error. (D) Relative translation error.

For the experimental evaluation, we used the UR5e robot arm with a Microsoft AzureKinect camera mounted on the last link for 2D image acquisition. During the experiment, the robot arm moved to random positions and orientations, and the image of a stationary calibration pattern was captured by the camera from which the poses of the camera with respect to the world was calculated, while the robot poses were obtained from the robot pendant. This procedure was done for 100 different motions of the robot. Fig 8A and 8B show the span of the rotation and translation motions, respectively with a mean rotation of 44.7 deg and a mean translation of 350.6 mm. The rotation and translation parameters of the hand-eye transformation was calculated from the acquired data using each of the algorithms. The comparison of the rotation and translation errors for the different algorithms under this condition is shown in Fig 8C and 8D, respectively. The exact values of the results for the different algorithms can be found on S1 Table in the S1 File. Because of the absence of ground truth data for the comparison, the relative rotation *E*_*R*_ and translation *E*_*T*_ errors were used for the evaluation instead.

From Fig 8C, the rotation estimated from Method Tsai showed the highest error, while the separated methods of Method Park and Method Chou provided the best estimate of the rotation based on the relative rotation error, with Method Park slightly outperforming Method Chou. All three simultaneous methods had similar rotation performance but were better than the Method Tsai, with the Method Daniilidis slightly outperforming the others. For the translation error (Fig 8D, all three simultaneous methods outperformed the separated methods with Method Daniilidis showing the best translation estimate. Method Tsai showed the best translation estimate among the separated methods, followed by Method Chou and then Method Park.

#### Effect of robot motion range

**Fig 9:**
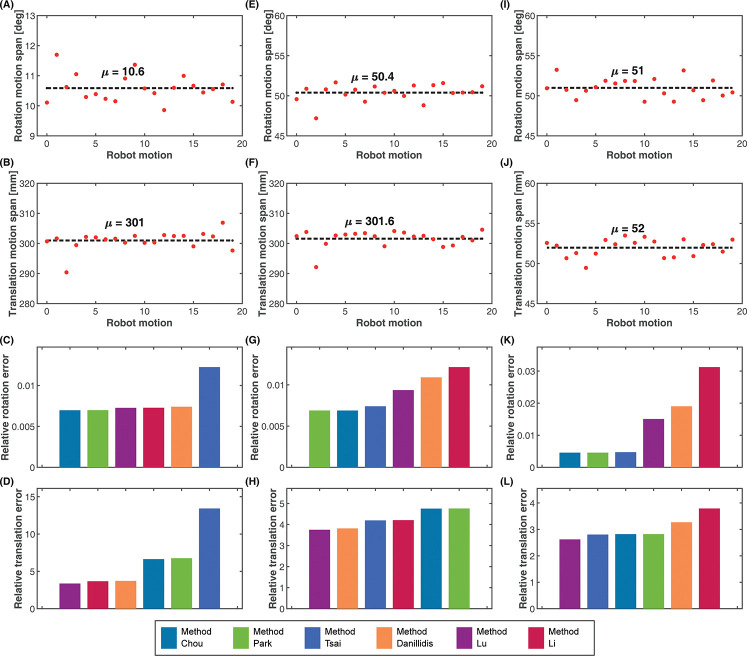
Motion range experiment. Experiment 1. Low rotation and high translation motion span. (A) Rotation motion span, mean *μ* = 10.6 deg. (B) Translation motion span, mean *μ* = 301.0 mm. (C) Rotation estimates. (D) Translation estimates. Experiment 2: High rotation and high translation motion span. (E) Rotation motion span, mean *μ* = 50.4 deg. (F) Translation motion span, mean *μ* = 301.6 mm. (G) Rotation estimates. (H) Translation estimates. Experiment 3: High rotation, low translation motion span. (I) Rotation motion span, mean *μ* = 51.0 deg. (J) Translation motion span, mean *μ* = 52.0 mm. (K) Rotation estimates. (L) Translation estimates.

The aim of the experiment is to observe how the rotation and translation motions in isolation affect the calibration accuracy for the candidate algorithms. We carried out three calibration operations with the real robot. For each calibration operation, we restricted the range of the translation and rotation motions to different values. The actual motion range for each motion was allowed to vary a little from the chosen span value. Table 2 shows the mean translation and rotation motion ranges for each of the experiments. Experiments 1 and 2 describe a change in the rotation motion span with a fixed translation motion span, while Experiments 2 and 3 describe a change in the translation motion span with a fixed rotation motion span. The results of the experiments are shown in Fig 9. The exact values of the results for the different algorithms can be found on S2 Table in the S1 File.

**Table 2:** Motion range experiments.

Experiments	Mean rotation motion range (deg)	Mean translation motion range (mm)
Experiment 1	10.6	301.0
Experiment 2	50.4	301.6
Experiment 3	51.0	52.0

From the results of Experiments 1 and 2, as shown in Fig 9A and 9E, respectively, the rotation range increased from 10.6 deg to 50.4 deg while the translation range remained close to 301 mm from Fig 9B and 9F. During these conditions, the rotation errors for Method Chou and Method Park remained relatively the same, with marginal improvements as shown in Fig 9C and 9G. However, a significant improvement in the rotation accuracy was observed for Method Tsai as the rotation motion range increased with constant translation motion range. Conversely, an increase in the error of the rotation estimate was observed for all the simultaneous methods as the rotation range increased with a fixed translation range. This increase in the rotation error was more pronounced in Method Li. In terms of the translation estimates, from Fig 9D and 9H, increasing the rotation range also improved the accuracy of the translation estimates for all the separated methods. The increment was also more noticeable in Method Tsai than in Method Chou and Method Park. However, just like the rotation estimates, Fig 9D and 9H shows that the accuracy of the translation estimates for all the simultaneous methods decreased when the rotation range was increased at the fixed translation range, with Method Li performing the worst.

The results in Fig 9F and 9J, respectively, show a decrease in the translation range from 301.6 mm in Experiment 2 to 52 mm in Experiment 3, while the rotation range from Fig 9E and 9I remains fixed at about 50 deg. From these results, it can be observed that as the translation motion range decreased from 301.6mm to 52 mm and at a constraint rotation motion range, the accuracy of the rotation estimates for the simultaneous methods decreased as seen in Fig 9G and 9K, with a more pronounced decrease in Method Li. The separated methods on the other hand experienced an increase in the accuracy of their rotation estimates when the translation range decreased with a fixed rotation range.

Observations from the translation estimates in Fig 9H and 9L show that the translation errors of all the calibration methods increased when the translation range was increased with a fixed rotation range. However, all the separated methods had a much higher increment in their translation errors than and even surpassed the translation errors of all the simultaneous methods. This suggests that the performance of the translation estimates of all the simultaneous methods improved much better than all the separated methods.

### Simulation versus real experiment

The use of simulated data allows deeper insight into the evaluation of the behaviours of the different algorithms, which may not be possible with the use of real data from experimentation. For instance, the availability of ground truth data. However, with real data from experiments, there is the advantage of capturing the true dynamics of the system under test, which may not be completely possible via simulation.

For the evaluations in this study, the availability of ground truth data during simulation allows the comparison based on absolute errors in rotation and translation, which ideally should be the better evaluation metrics. On the other hand, because ground truth data is not available for the real experiment, relative rotation and translation errors were used for the evaluation. Hence there is the expectation of discrepancies in the evaluations, for example, the relative difference in observed simulation errors between the evaluated algorithms compared with the real experiment. We have also used the relative errors for the evaluation of the estimated errors based on the robot motion range to validate the simulation study with the experiment in the absence of ground truth data. Furthermore, as we observed in the simulation tests, the rotation and translation errors depend on a number of factors. These factors have been evaluated at specific values and ranges during simulation. The total rotation and translation errors as seen from the experimental evaluation are a combination of the errors from each of these factors, which are largely unknown.

## Conclusion

This study comparatively evaluates the accuracy of some of the common hand-eye calibration algorithms based on several factors: the use of simulated datasets and real datasets from experimentation with a physical robot. The result of the comparative study sheds light on how different factors affect the accuracy of estimates based on these methods.

Firstly, the number of robot motions used during the calibration is critical to the level of accuracy of the estimated hand-eye parameters in the presence of noise. While it has been established in the literature that a minimum of two relative motions (or 3 robot poses) are necessary for the estimation, increasing the number of motions increases the accuracy level. However, as the number of robot motions increases, the improvement in the accuracy achieved becomes minimal. Moreover, the number of robot motions used would impact the execution time, complexity, and computation cost of the different algorithms. From our study, Method Daniilidis incurred the highest computation cost when the number of robot motions was low, while Method Li incurred the highest computation cost when the number of robot motions was high. Method Park appeared to be the most computationally efficient method.

The results from this study show that Method Tsai was extremely sensitive to rotation noise, and its estimated parameters are only comparable to others in very low noise conditions. Furthermore, the noise in the rotation and translation motions affect the rotation and translation estimates in different ways in all the evaluated hand-eye calibration methods. While the quality of the estimated translation depends on the estimated rotation parameter for the separated methods, estimating the rotation and translation parameters together as in the simultaneous methods resulted in noise transfer between both parameters. Judging from the combined effect of rotation and translation noise, the Methods of Daniilidis, Park and Chou appeared to be the most reliable methods of all the algorithms evaluated as they consistently showed greater performance from all the simulation studies. However, while Method Daniilidis showed slightly better performance at lower rotation and translation noise levels, its performance degrades below that of Methods Park and Chou as the noise levels increases. Methods Lu and Li consistently showed roughly similar performance, however Method Lu appeared more suited to lower noise level than Method Li.

The range of motion of the robot during calibration was also shown to have a significant impact on the performance of the calibration algorithms. As shown in the simulation study and validated by the experiments with the real robot, the separated solution methods of Method Chou, Method Park and Method Tsai performed better at higher rotation and lower translation motions of the robot. However, for the simultaneous methods of Method Li, Method Daniilidis and Method Lu, better performance can be achieved by using lower rotation and translation motions of the robot.

It is important to note that while all the separated methods showed similar performance during the experiments on the effect of motion range, and likewise the simultaneous methods, the conclusions of this study were peculiar to the methods surveyed and not the solution class, which is by no means exhaustive in this study. The results of the effect of motion clearly showed that the accuracy of a hand-eye calibration algorithm would vary substantially with different ranges of motions of the robot during calibration. As such, this factor should be taken into consideration when benchmarking a particular algorithm against other algorithms.

While the factors affecting the accuracy of the hand-eye calibration have been established in literature, the focus in previous works has been on the effect of noise on the calibration accuracy. Moreover, evaluation of algorithms put more emphasis on the effect of translation and rotation combined. The work in this paper goes further to show the specific impact of the rotation noise and the translation noise on the calibration accuracy, as well as the role the range of rotation and translation motions used during the calibration play on the calibration accuracy. This paper evaluates how these factors affect the different algorithms comparatively using similar datasets and test points. Hence, depending on the application constraints, a user can select a suitable algorithm with the implementation details given.

While authors of different hand-eye calibration algorithms have documented their sensitivities to noise, not much research has been done to assess and mitigate the impact of motion range on hand-eye calibration. This may be especially important in space applications where the size and mass of payload is very critical. As such, this path of research presents an interesting topic for the research community to address. Furthermore, the insight provided from the analysis of impact of the rotation and translation noise in isolation raises the question, “are there some advantage to be gained in restricting the robot motion to either of these motions?” This can be answered by carrying out further research.

## Supporting information

S1 Appendix Supporting material: S tables, S links, S video. (DOCX)

S1 File (DOCX)

## References

[1] IFR. IFR forecast: 1.7 million new robots to transform the world´s factories by 2020. In: IFR International Federation of Robotics [Internet]. [cited 22 Jun 2022]. Available: https://ifr.org/news/ifr-forecast-1.7-million-new-robots-to-transform-the-worlds-factories-by-20/.

[2] Misimi Øye, Eilertsen Mathiassen. GRIBBOT–Robotic 3D vision-guided harvesting of chicken fillets. and Electronics in …. Available: https://www.sciencedirect.com/science/article/pii/S0168169915003701?casa_token=y78yeTY1z-4AAAAA:ttA3bhnaJZNo-vrrtUgjsy1vrio1CyRm_BpHUegOzKBZ6GxqiL7KyBH5ApTBQhBDYPFSVdZ9lbY.

[3] Bjørlykhaug E, Egeland O. Vision System for Quality Assessment of Robotic Cleaning of Fish Processing Plants Using CNN. IEEE Access. 2019;7: 71675–71685.

[4] Faisal M, Alsulaiman M, Arafah M, Mekhtiche MA. IHDS: Intelligent Harvesting Decision System for Date Fruit Based on Maturity Stage Using Deep Learning and Computer Vision. IEEE Access. 2020;8: 167985–167997.

[5] Wan J, Tang S, Li D, Imran M, Zhang C, Liu C, et al. Reconfigurable Smart Factory for Drug Packing in Healthcare Industry 4.0. IEEE Trans Ind Inf. 2019;15: 507–516.

[6] Park M, Le T-A, Eizad A, Yoon J. A Novel Shared Guidance Scheme for Intelligent Haptic Interaction Based Swarm Control of Magnetic Nanoparticles in Blood Vessels. IEEE Access. 2020;8: 106714–106725.

[7] Jayaweera N, Webb P, Johnson C. Measurement assisted robotic assembly of fabricated aero‐engine components. Assembly Automation. 2010;30: 56–65.

[8] Li Z, Suntharasantic S, Bai S, Chirarattananon P. Aeromechanic Models for Flapping-Wing Robots With Passive Hinges in the Presence of Frontal Winds. IEEE Access. 2018;6: 53890–53906.

[9] Wu L, Li H, Li Y, Li C. Position Tracking Control of Tailsitter VTOL UAV With Bounded Thrust-Vectoring Propulsion System. IEEE Access. 2019;7: 137054–137064.

[10] Li Z, Li S, Luo X. An overview of calibration technology of industrial robots. IEEE/CAA j autom sin. 2021;8: 23–36.

[11] Bentaleb T, Iqbal J. On the improvement of calibration accuracy of parallel robots–modeling and optimization. Journal of Theoretical and Applied Mechanics. 2020. pp. 261–272. doi: 10.15632/jtam-pl/115863

[12] Qi W, Li F, Zhenzhong L. Review on camera calibration. 2010 Chinese Control and Decision Conference. 2010. pp. 3354–3358.

[13] Gong X, Lv Y, Xu X, Jiang Z, Sun Z. High-Precision Calibration of Omnidirectional Camera Using an Iterative Method. IEEE Access. undefined 2019;7: 152179–152186.

[14] Shiu YC, Ahmad S. Calibration of wrist-mounted robotic sensors by solving homogeneous transform equations of the form AX = XB. IEEE Transactions on Robotics and Automation. 1989. pp. 16–29. doi: 10.1109/70.88014

[15] Li A, Wang L, Wu D. Simultaneous robot-world and hand-eye calibration using dual-quaternions and Kronecker product. Int J Physic Sci. 2010;5: 1530–1536.

[16] Aghakhani N, Geravand M, Shahriari N, Vendittelli M, Oriolo G. Task control with remote center of motion constraint for minimally invasive robotic surgery. 2013 IEEE International Conference on Robotics and Automation. 2013. pp. 5807–5812.

[17] Gu Y-K, Li W-F, Zhang J, Qiu G-Q. Effects of Wear, Backlash, and Bearing Clearance on Dynamic Characteristics of a Spur Gear System. IEEE Access. 2019;7: 117639–117651.

[18] Mavroidis C, Dubowsky S, Drouet P, Hintersteiner J, Flanz J. A systematic error analysis of robotic manipulators: application to a high performance medical robot. Proceedings of International Conference on Robotics and Automation. 1997, pp. 980–985 vol.2.

[19] Zannatha J, Limon R. Forward and Inverse Kinematics for a Small-Sized Humanoid Robot, 2009 International Conference on Electrical, Communications, and Computers, 2009, pp. 111–118.

[20] Singh TP, Suresh P, Chandan S. Forward and inverse kinematic analysis of robotic manipulators. International Research Journal of Engineering and Technology (IRJET). 2017;4: 1459–1468.

[21] Lu XX. A Review of Solutions for Perspective-n-Point Problem in Camera Pose Estimation. J Phys Conf Ser. 2018;1087: 052009.

[22] Yi G, Jianxin L, Hangping Q, Bo W. Survey of structure from motion. Proceedings of 2014 International Conference on Cloud Computing and Internet of Things. 2014. pp. 72–76.

[23] Sun C, Sun P, Wang P. An Improvement of Pose Measurement Method Using Global Control Points Calibration. PLoS One. 2015;10: e0133905. doi: 10.1371/journal.pone.0133905 26207825 PMC4514832

[24] Gai S, Da F, Fang X. A Novel Camera Calibration Method Based on Polar Coordinate. PLoS One. 2016;11: e0165487. doi: 10.1371/journal.pone.0165487 27798651 PMC5087901

[25] Kim YY, Jeong M-H, Kang DJ. Mobile robot calibration. IECON 2013 - 39th Annual Conference of the IEEE Industrial Electronics Society. 2013. pp. 5504–5506.

[26] Lee J-W, Park G-T, Shin J-S, Woo J-W. Industrial robot calibration method using denavit—Hatenberg parameters. 2017 17th International Conference on Control, Automation and Systems (ICCAS). IEEE; 2017. pp. 1834–1837.

[27] Hsiao J-C, Shivam K, Lu I-F, Kam T-Y. Positioning Accuracy Improvement of Industrial Robots Considering Configuration and Payload Effects Via a Hybrid Calibration Approach. IEEE Access. undefined 2020;8: 228992–229005.

[28] Jafri SRuN, Shamim S, Faraz SM, Ahmed A, Yasir SM, Iqbal J (2022) Characterization and calibration of multiple 2D laser scanners. PLoS ONE 17(7): e0272063. doi: 10.1371/journal.pone.0272063 35900977 PMC9333273

[29] Zhou B, Chen Z, Liu Q. An Efficient Solution to the Perspective-n-Point Problem for Camera With Unknown Focal Length. IEEE Access. 2020;8: 162838–162846.

[30] Tsai RY, Lenz RK. A new technique for fully autonomous and efficient 3D robotics hand/eye calibration. IEEE Transactions on Robotics and Automation. 1989. pp. 345–358. doi: 10.1109/70.34770

[31] Chou JCK, Kamel M. Finding the Position and Orientation of a Sensor on a Robot Manipulator Using Quaternions. Int J Rob Res. 1991;10: 240–254.

[32] Park FC, Martin BJ. Robot sensor calibration: solving AX = XB on the Euclidean group. IEEE Trans Rob Autom. 1994;10: 717–721.

[33] Chen. A screw motion approach to uniqueness analysis of head-eye geometry. Proceedings 1991 IEEE Computer Society Conference on Computer Vision and Pattern Recognition. 1991. p. 145,146,147,148,149,150,151.

[34] Zhao Z, Liu Y. Hand-Eye Calibration Based on Screw Motions. 18th International Conference on Pattern Recognition (ICPR’06). 2006. pp. 1022–1026.

[35] Daniilidis K, Bayro-Corrochano E. The dual quaternion approach to hand-eye calibration. Proceedings of 13th International Conference on Pattern Recognition. 1996. doi: 10.1109/icpr.1996.546041

[36] Lu Y-C, Chou JCK. Eight-space quaternion approach for robotic hand-eye calibration. 1995 IEEE International Conference on Systems, Man and Cybernetics Intelligent Systems for the 21st Century. 1995. pp. 3316–3321 vol.4.

[37] Li W, Dong M, Lu N, Lou X, Sun P. Simultaneous Robot–World and Hand–Eye Calibration without a Calibration Object. Sensors. 2018. p. 3949. doi: 10.3390/s18113949 30445680 PMC6263626

[38] Qiu S, Wang M, Kermani MR. A New Formulation for Hand–Eye Calibrations as Point-Set Matching. IEEE Trans Instrum Meas. 2020;69: 6490–6498.

[39] Wu J, Sun Y, Wang M, Liu M. Hand-Eye Calibration: 4-D Procrustes Analysis Approach. IEEE Trans Instrum Meas. 2020;69: 2966–2981.

[40] Liu J, Wu J, Li X. Robust and Accurate Hand–Eye Calibration Method Based on Schur Matric Decomposition. Sensors. 2019;19: 4490. doi: 10.3390/s19204490 31623249 PMC6832585

[41] Tabb A, Ahmad Yousef KM. Solving the robot-world hand-eye(s) calibration problem with iterative methods. Machine Vision and Applications. 2017. pp. 569–590. doi: 10.1007/s00138-017-0841-7

[42] OpenCV: Camera Calibration and 3D Reconstruction. [cited 8 Dec 2021]. Available: https://docs.opencv.org/3.4.15/d9/d0c/group__calib3d.html.

[43] Koide K, Menegatti E. General Hand–Eye Calibration Based on Reprojection Error Minimization. IEEE Robotics and Automation Letters. 2019;4: 1021–1028.

[44] Zhao Z. Simultaneous robot-world and hand-eye calibration by the alternative linear programming. Pattern Recognit Lett. 2019;127: 174–180.

[45] Shah M. Solving the Robot-World/Hand-Eye Calibration Problem Using the Kronecker Product. J Mech Robot. 2013;5. doi: 10.1115/1.4024473

[46] Li H, Ma Q, Wang T, Chirikjian GS. Simultaneous hand-eye and robot-world calibration by solving the ax = yb problem without correspondence. IEEE Robotics and Automation Letters. 2015;1: 145–152.

