# Supplementary material for: Accuracy evaluation of hand-eye calibration techniques for vision-guided robots
Source: PLoS One. 2022 Oct 19;17(10):e0273261. doi: 10.1371/journal.pone.0273261 (PMC9581431; doi:10.1371/journal.pone.0273261)
Supplement: S1 Appendix — (DOCX) [file pone.0273261.s001.docx]

# **SI Appendix: Supporting Material**

**Accuracy evaluation of hand-eye calibration techniques for vision-guided robots**

Ikenna Enebuse^1^, Babul KSM Kader Ibrahim^2^, Mathias Foo^3^, Ranveer S. Matharu^1^ and Hafiz Ahmed^4^*

**^1^** Centre for Future Transport and Cities, Coventry University, Coventry, United Kingdom

**^2^** School of Mechanical, Aerospace and Automotive Engineering, Coventry University, Coventry, United Kingdom

**^3^** School of Engineering, University of Warwick, Coventry, United Kingdom

**^4^** Nuclear Futures Institute, Bangor University, Bangor, United Kingdom

*Corresponding author

Email: hafiz.ahmed@bangor.ac.uk (HA)

**Supplementary Link**

**S1 Link.** **Link to python implementation of algorithms and dataset.**

<https://github.com/ienebuse/hand-eye-calibration>

**Supplementary Tables**

**S1 Table. Table of rotation and translation error from experiment according to Fig 8.**

| Algorithms | Rotation error (deg) | Translation error (mm) |
| --- | --- | --- |
| Method Chou | 0.0062 | 6.2315 |
| Method Park | 0.0062 | 6.4441 |
| Method Tsai | 0.013 | 4.9577 |
| Method Daniilidis | 0.0095 | 4.3481 |
| Method Lu | 0.0097 | 4.3694 |
| Method Li | 0.0099 | 4.6135 |

**S2 Table. Table of experimental results from motion range study according to Fig 9.**

| Algorithms | Rotation range: 10.6 deg  Translation range: 301 mm | | Rotation range: 50.4 deg  Translation range: 301.6 mm | | Rotation range: 51 deg  Translation range: 52 mm | |
| --- | --- | --- | --- | --- | --- | --- |
|  | Rotation error | Translation error | Rotation error | Translation error | Rotation error | Translation error |
| Method Chou | 0.007 | 6.6416 | 0.0069 | 4.7555 | 0.0046 | 2.8222 |
| Method Park | 0.007 | 6.7641 | 0.0069 | 4.7616 | 0.0046 | 2.8228 |
| Method Tsai | 0.0122 | 13.4277 | 0.0074 | 4.1929 | 0.0047 | 2.8058 |
| Method Daniilidis | 0.0074 | 3.723 | 0.0109 | 3.8138 | 0.019 | 3.2697 |
| Method Lu | 0.0073 | 3.3761 | 0.0094 | 3.748 | 0.0151 | 2.6211 |
| Method Li | 0.0073 | 3.677 | 0.0122 | 4.2053 | 0.0312 | 3.7925 |

**Supplementary Video**

**S1 Video.** **Video of calibration demo**

https://github.com/ienebuse/hand-eye-calibration/blob/main/calibration_demo.mp4
